# Mitochondrial dysfunction in vascular endothelial cells and its role in atherosclerosis

**DOI:** 10.3389/fphys.2022.1084604

**Published:** 2022-12-20

**Authors:** Kai Qu, Fang Yan, Xian Qin, Kun Zhang, Wen He, Mingqing Dong, Guicheng Wu

**Affiliations:** ^1^ Clinical Research Center for Endocrinology and Metabolic Diseases, Chongqing University Three Gorges Hospital, Chongqing, China; ^2^ College of Bioengineering Chongqing University, Chongqing, China; ^3^ Department of Geriatrics, Geriatric Diseases Institute of Chengdu, Chengdu Fifth People’s Hospital, Chengdu, Sichuan, China; ^4^ Center for Medicine Research and Translation, Chengdu Fifth People’s Hospital, Chengdu, Sichuan, China; ^5^ Department of Geriatrics, Clinical trial center, Chengdu Fifth People’s Hospital, Chengdu, Sichuan, China

**Keywords:** mitochondrial dysfunction, endothelial cells, atherosclerosis, mitochondrial ROS, mitophagy

## Abstract

The mitochondria are essential organelles that generate large amounts of ATP *via* the electron transport chain (ECT). Mitochondrial dysfunction causes reactive oxygen species accumulation, energy stress, and cell death. Endothelial mitochondrial dysfunction is an important factor causing abnormal function of the endothelium, which plays a central role during atherosclerosis development. Atherosclerosis-related risk factors, including high glucose levels, hypertension, ischemia, hypoxia, and diabetes, promote mitochondrial dysfunction in endothelial cells. This review summarizes the physiological and pathophysiological roles of endothelial mitochondria in endothelial function and atherosclerosis.

## Introduction

Cardiovascular diseases (CVDs), including angina, myocardial infarction, and ischemic stroke, are the leading causes of morbidity and mortality worldwide (Roth et al., 2017; D. Zhao D et al., 2019). Atherosclerosis is a chronic inflammatory disease, that is, the major factor of these diseases (Bjorkegren and Lusis, 2022). The mitochondria are vital organelles in eukaryotic cells that govern energy transformation, where they generate large amounts of ATP for cellular various metabolic processes, such as tricarboxylic acid cycle (TCA) and oxidative phosphorylation (Heine and Hood, 2020). The mitochondria are also involved in other cellular processes, such as apoptosis, proliferation, ion homeostasis, inflammation, and oxidative stress (Gorman et al., 2016). Recently studies have shown that mitochondrial damage and dysfunction are important factors in the initiation and progression of atherosclerosis (Peng et al., 2019; Salnikova et al., 2021). The pro-atherosclerotic role of mitochondrial damage and dysfunction has been well elucidated in SMCs and monocytes (Duan et al., 2022; Dumont et al., 2021; Huynh and Heo, 2021). However, the crucial roles of endothelial mitochondrial damage and dysfunction in the progression of atherosclerosis have not received much attention. Thus, this review summarizes the physiological and pathophysiological roles of endothelial mitochondria in endothelial function and atherosclerosis.

## The key roles of endothelial cells in atherosclerosis

The development of atherosclerotic plaques is a complex process involving many steps and the interaction of systemic and local factors. Initiation of atherosclerosis is activated by endothelium activation followed by endothelial dysfunction, fatty streak formation, fibrous plaque formation, advanced plaque formation and plaque rupture. Advanced atherosclerotic plaques can protrude into the arterial lumen and occupy the lumen space, which hinders blood flow and leads to tissue ischemia (Jebari-Benslaiman et al., 2022). Unstable atherosclerotic plaques can rupture, resulting in thrombosis and blood flow interruption (Bjorkegren and Lusis, 2022). Altogether, these processes cause cardiovascular complications which are as the main cause of death worldwide.

The vascular endothelium is a monolayer formed by endothelial cells (ECs), which covers the inner wall of all blood vessels, which is directly stimulated by cardiovascular risk factors from circulation (Jebari-Benslaiman et al., 2022). Vascular ECs play a critical role in the initiation and progression of atherosclerosis (Souilhol et al., 2018). Endothelial dysfunction is caused by a various cardiovascular risk factors and exacerbates the progression of atherosclerotic plaques (Medina-Leyte et al., 2021). On the one hand, activated ECs trigger local inflammation by inducing the expression of inflammatory cytokines (such as interleukin (IL)-8, monocyte chemoattractant protein-1) and adhesion molecules [such as intercellular adhesion molecule-1 (ICAM-1) and vascular cell adhesion molecule-1 (VCAM-1)] and attracting monocytes to bind to the activated endothelial monolayer; on the other hand, activated ECs accelerate the accumulation of lipids (specifically, plasma low-density lipoprotein, LDL), immune cells, SMCs, fibroblasts, and extracellular matrix in the subendothelial space and drive atherogenesis (Libby et al., 2019). Disorders of ECs represent an important pathological mechanism of atherosclerosis, especially in an early step in the development of atherosclerosis. Atherosclerosis-related risk factors induce mitochondrial dysfunction in ECs, which may be the main cause of atherosclerosis-related risk factor-induced endothelial disorders.

## Excessive endothelial mitochondrial ROS and atherosclerosis

### Mitochondrial ROS production

**FIGURE 1:**
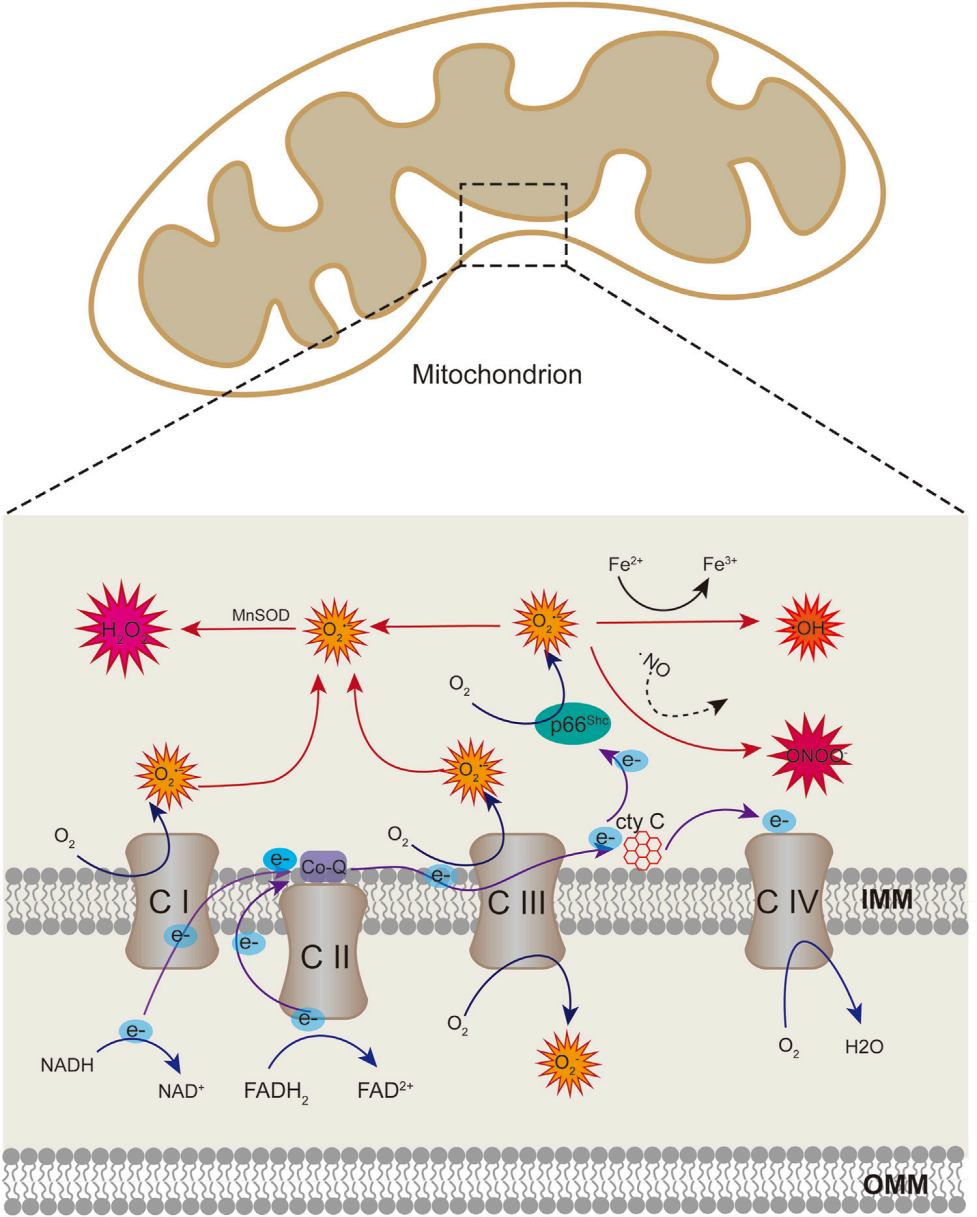
Schematic showing mitochondrial sources of ROS. Superoxide anion can be produced at the sites of complex I and III of the respiratory chain through electron transport. When cytochrome c transfers electrons from complex III to complex IV, p66Shc can subtract complex III-derived electrons from cytochrome c, leading to cytochrome c oxidized and producing superoxide anion. Superoxide anion can be converted to hydrogen peroxide (H2O2) by superoxide dismutase enzymes (SOD). Superoxide can also react with nitric oxide (NO) to produce peroxinitrite (ONOO). Hydrogen peroxide generate hydroxyl radicals in the presence of transition metals.

ROS include superoxide anion (O_2_^•−^), hydrogen peroxide (H_2_O_2_), hydroxyl radical (OH), and peroxynitrite (ONOO^−^). The free radical superoxide anion, which is responsible for the formation of other reactive species in the vascular endothelium, is the first to be generated. The mitochondria are the primary source of ROS *via* electron transport chain (ETC) in eukaryotic cells (Bugger and Pfeil, 2020). Superoxide anion is essentially produced in the mitochondrial complex I and III because of electron leakage from the ETC. Iron-sulfur centers of ETC can be oxidized by mitochondrial ROS (mtROS), causing the functional damage of ETC complexes and exacerbating the production of ROS (Incalza et al., 2018). ETC complexes are the major source of ROS generation in the mitochondria (Shadel and Horvath, 2015). However, in the endothelium, more than 80% of ATP comes from glycolysis rather than ETC, then there may be a decline in the number of ROS from mitochondria (Quintero et al., 2006). In addition to ETC, several proteins may also generate ROS in the mitochondria. p66Shc is a protein with 66 kDa which is localized in the intermembrane space; it acts as a redox enzyme to generate ROS by oxidating cytochrome c and subsequently reduce molecular oxygen to O_2_^•−^(Figure 1) (Giorgio et al., 2005). NADPH oxidases (NOX) is another system of ROS generation *via* transporting electrons to oxygen from NADPH to produce superoxide free radical (Bedard and Krause, 2007). NOX4 could localise in mitochondria in macrophage and kidney cortex (Block et al., 2009; Moon et al., 2016), which contributes to the mtROS pool. Besides, mitochondria-localized uncoupled eNOS also increase mtROS generation in ECs (Chen et al., 2014).

### The vascular risk factors cause excessive mitochondrial ROS production in endothelium

The mitochondria produce excessive ROS because of various reasons (Table 1). Mitochondrial fission is an important factor of mtROS overproduction. The high glucose-induced ROS production was accompanied by a marked change in mitochondrial morphology. Mitochondrial fission is required for increased production of mtROS in hyperglycemic conditions or ischemia (I)/reperfusion (RP)-induced mtROS production in ECs (Giedt et al., 2012b; Liu et al., 2019; Yu et al., 2006). Dynamin-related protein 1 (DRP1) is a key protein to mediate mitochondrial fission. Inhibition of DRP1 phosphorylation by P110, a DRP1-specific peptide inhibitor, attenuated LPS-induced mtROS (Fu et al., 2021).

**TABLE 1:** The induced factors of excessive mtROS in endothelial cells.

Induced factors	Pathway	Reference
High glucose/Diabetes	I Increasing voltage gradient	Cho et al. (2013); Fiorentino et al. (2013); Guo et al. (2018); Rolo and Palmeira (2006); Yu et al. (2006)
	II Protein ubiquitination of SOD2	
	III Inducing mitochondrial fragmentation/fission	
Angiotensin II/Hypertension	I NOX2/NOX4-PKC-p66Shc axis	Dikalov et al. (2014); Doughan et al. (2008); Kim et al. (2017); Takac et al. (2011)
	II Inhibiting the activity of mitochondrial electron transport chain complex I, leading to a reduction of mitochondrial oxidative capacity	
Mitochondria fission	I Phosphorylation of DRP1	Cali and Szabadkai, (2018)
Parathyroid hormone/Hyperthyroidism	I Regulation of serum Ca^2+^ concentrations	Gambardella et al. (2018); Khundmiri et al. (2016)
ROS/MtROS	I oxidative damage of the mitochondrial respiratory complexes	Daiber (2010); van der Loo et al. (2000)
	II Inactivation of the endogenous antioxidant mitochondrial SOD	
M1 macrophage-derived exosomes	I Increasing mitochondrial fragmentation through microRNA-155 targeting SOCS6, leading to phosphorylation of DRP1	Ge et al. (2021); Lin et al. (2013)
LPS	I Phosphorylation of DRP1	Fu et al. (2021)
MtDMAPs (Trimethylamine N-oxide)	I Activating PGC-1alpha	Fujisawa et al. (2009)
Doxorubicin	Unknown	Clayton et al. (2020)
Ox-LDL	Unknown	P. Li P et al. (2021)

Blood-spinal-cord-barrier disruption after spinal cord injury leads to the infiltration of numerous peripheral macrophages into injured areas and accumulation around newborn vessels (Lee et al., 2012). In the process, exosomes from M1-polarized bone marrow-derived macrophages induce mitochondrial dysfunction and ROS accumulation of vascular ECs by microRNA-155 to target suppressor of cytokine signaling 6 (SOCS6) (Ge et al., 2021). SOCS6 inhibits the phosphorylation of DRP1, thereby promoting its mitochondrial translocation to participate in mitochondrial fragmentation (Lin et al., 2013).

High glucose (50 mM) levels induce mtROS overproduction (Yu et al., 2006). Normally, protons are extruded into the intermembrane space in the mitochondrial ETC, which creates a proton gradient to drive ATP synthase (complex V) back through the inner membrane to the matrix (Guo et al., 2018). When cells are within high intracellular glucose concentration, more electron donors (NADH and FADH2) into the ETC due to more glucose-derived pyruvate are oxidized in the TCA cycle. As a result, the voltage gradient across the mitochondrial membrane increases until a critical threshold is reached. At this point, electron transfer within complex III is blocked, causing electrons to return to Coenzyme Q, which donates electrons to oxygen molecules one at a time and induces superoxide production (Fiorentino et al., 2013; Rolo and Palmeira, 2006). In cultured primary arterial ECs, intracellular hyperglycemia raises the voltage across the mitochondrial membrane beyond the critical threshold required to increase superoxide formation. High-glucose treatment increases protein ubiquitination of superoxide dismutase 2 (SOD2) leading to SOD2 protein degradation followed by a decrease in protein level. SOD2 antioxidative activity is lower accompanied by a lower protein level, which also causes high glucose-induced ROS overproduction (Cho et al., 2013). In addition, mitochondrial fragmentation/fission is necessary for high glucose-induced respiration and excessive ROS production (Yu et al., 2006).

Hyperthyroidism promotes the generation of mtROS. However, few studies analyzed the effects of thyroid hormone on the mtROS production of the endothelium. Furthermore, flow-mediated dilation and intima-media thickness can be reversed by parathyroidectomy (Yankouskaya and Snezhitskiy, 2014), suggesting that the endothelium is a key target of parathyroid hormone, a known regulator of serum Ca^2+^ concentrations (Khundmiri et al., 2016). The parathyroid hormone induces mtROS production in a calcium-dependent manner (Gambardella et al., 2018).

Angiotensin II (Ang II) promotes the production of vascular endothelial ROS in the cellular cytoplasm and mitochondria. Inhibition of NOX2 by apocynin completely prevents Ang II-induced mitochondrial dysfunction and attenuates mtROS production Ang II induces mitochondrial dysfunction *via* a PKC-dependent pathway by activating the endothelial NOX2. In this process, mitochondrial PKCε is an important downstream target of NOX2, and subsequently PKCε activates mitochondrial ATP-sensitive potassium channels, which trigger mitochondrial reverse electron transfer and leading to O_2_^•−^ generation (Dikalov et al., 2014). Furthermore, mtROS overproduction is as a secondarily consequence of NOXs activation-induced cytoplasmic NO˙ and O_2_^•−^ generation (Doughan et al., 2008). NOX4 is responsible for the basal production of H_2_O_2_ (Takac et al., 2011). Exogenous H_2_O_2_ or overexpression of NOX4, which produces H_2_O_2_, increases mtROS production because NOX2 senses NOX4-derived H_2_O_2_ to promote mtROS production *via* the phosphorylation of p66Shc at the Ser36 site through mitochondrial PKC (Kim et al., 2017). However, another study reported that NOX4 partially co-localizes with the mitochondria. Thus, it may also be considered a source of mitochondrial ROS. Mitochondrial NOX4 specifically inhibits the activity of mitochondrial electron transport chain complex I, leading to a reduction of mitochondrial oxidative capacity and acceleration of respiratory chain-mediated ROS generation (Koziel et al., 2013).

The mitochondria are a source of ROS and also a target of excess ROS. Excessive ROS can elicit oxidative damage to the mitochondrial respiratory complexes and inactivation of the endogenous antioxidant mitochondrial SOD, which amplify mtROS production and reduce the consumption by SOD (Daiber, 2010; Doughan et al., 2008; van der Loo et al., 2000).

In addition to the above mentioned, several other cardiovascular risk factors, including oxidized low-density lipoprotein (P. Li P et al., 2021), LPS(Fu et al., 2021) and disturbed flow (Hong et al., 2022), can also induce an increase of mtROS levels in ECs. The overproduction of mtROS is also induced by several small molecular compounds, including trimethylamine-N-oxide (Wu et al., 2020), doxorubicin (Clayton et al., 2020), thiazolidinediones (Fujisawa et al., 2009), urea (D'Apolito et al., 2018) in ECs.

### Excessive mitochondrial ROS production in endothelial cells accelerates atherosclerosis

Oxidative stress contributes to the development of inflammation in ECs, which is the initial process in the development of atherosclerosis (El et al., 2013). Mitochondrial DNA (mtDNA) plays an important role in ROS-induced endothelial inflammation (Shimada et al., 2012). MtDNA is more sensitive than genomic DNA to ROS-induced damage, because it is not protected by histones and limited repair capabilities (Wei and Lee, 2002). Due to the reactive nature of ROS, mtROS probably contributes to the high mutation rate of the mitochondrial genome and oxidative damage to the respiratory chain and lipid peroxidation (Heine and Hood, 2020; Park et al., 2021; Pinti et al., 2019; R. Wu R et al., 2018). Damaged mtDNA promotes the opening of mitochondrial permeability transition pore (MPTP), leading to outer membrane permeabilization. Apoptogenic protein release to the cytosol from the mitochondrial though the opening MPTP (Papu et al., 2019). In addition, malfunctioning of the mitochondrial genome is directly correlated with impaired mitochondrial physiology and depleted ATP-synthesis, which are accompanied by enhanced ROS formation and increased apoptosis (Salnikova et al., 2021). MtDNA is also released into the cytosol, particularly cytosolic oxidized mtDNA from oxidative damage mitochondria, which activates the NLRP3 inflammasome and consequently increases IL-1β release, which contributes to the adhesion and migration of monocytes into the intima (Shimada et al., 2012).

Pyroptosis is a programmed cell death characterized by plasma membrane rupture and followed by cellular contents and pro-inflammatory mediators release from rupture cell, playing a pro-atherosclerotic role (Hoseini et al., 2018). EC mitochondrial ROS accumulation induced by trimethylamine N-oxide (TMAO) and low shear stress accelerate the formation of atherosclerotic plaques by inducing ECs pyroptosis in ApoE-deficient mice fed with a high-fat diet (Chen C et al., 2021; Wu et al., 2020). TMAO is produced from the phosphatidylcholine metabolism of gut flora (Schwartz and Reaven, 2012), which promotes succinate dehydrogenase complex subunit B (SDHB) upregulation in vascular EC (Wu et al., 2020). SDHB is a member of the SDH family and a subunit of respiratory chain complex II (Mills et al., 2016). Its high expression increases mitochondrial ROS production, and further induces EC pyroptosis (Wu et al., 2020). Low shear stress plays key roles in the initiation and progression of atherosclerosis, which induce mtROS overproduction *via* upregulation of the SDHB expression, and further EC pyroptosis (Chen Y et al., 2021). In addition, ox-LDL and cholesterol crystals-induced (Yang et al., 2020) intracellular ROS and mtROS also caused EC pyroptosis (P. Li P et al., 2021; Zhaolin et al., 2019). EC pyroptosis can be inhibited *via* decreasing ROS by the ROS scavenger NAC in ECs exposed to TMAO, LPS and nicotine (Wu et al., 2020; X. Wu X et al., 2018; Zhao et al., 2021). These showed that ROS accumulation is required for EC pyroptosis. Inflammasome contain pattern-recognition receptors (PRR) that can be activated by damaged-associated molecular patterns (DAMPs) in inflammasome pathway-induced pyroptosis (Shi et al., 2017). Mitochondrial DAMPs can be released from oxidative damaged mitochondria by mtROS(Nakahira et al., 2015), which may bind to PRR, and activate subsequent pyroptosis cascade. However, the more detailed mechanism of mtROS overproduction-promoted pyroptosis is still unclear, especially in ECs.

The endothelial dysfunction is thus defined as an imbalance in the production of the vasodilator and vasoconstrictor factors, predisposing the vasculature toward a pro-thrombotic and pro-atherogenic phenotype, characterized by vasoconstrinction, leukocyte adhesion, platelet activation, mitogenesis, pro-oxidation, impaired coagulation, vascular inflammation, and thrombosis (Cyr et al., 2020; Incalza et al., 2018). The decreased synthesis, release and/or activity of endothelium-derived NO is one of the most important events that characterizes endothelial dysfunction (Siragusa et al., 2019). MtROS play an important role in normal physiological cell signaling to regulate important vascular homeostatic functions under basal conditions in various vascular beds. Upon exposure to cardiovascular risk factors, ECs produce excessive ROS that activate prothrombotic and proinflammatory pathways in the vascular endothelium and contribute to lipid peroxidation and oxidative modifications of proteins and nucleic acids, leading to endothelial dysfunction (Dikalov and Nazarewicz, 2013). Excessive ROS reduce NO bioavailability *via* the reaction of NO with superoxide to generate peroxynitrite, another potent oxidant (Liaudet et al., 2009). Excessive peroxynitrite generation induces protein nitration and broadly contributes to cellular nitrosative and oxidative stress and uncouples endothelial nitric oxide synthase (eNOS) (Cassuto et al., 2014; Diers et al., 2013; Radi, 2018). In ROS-mediated endothelial dysfunction, BH4 is oxidized to BH2, which cannot function as a cofactor of eNOS, causing eNOS uncoupling and ROS generation (Dikalova et al., 2016). Thus, eNOS uncoupling-derived ROS further oxidize BH4 to BH2, exacerbating Endothelial dysfunction.

Damaged endothelial barrier and consequently leukocyte transmigration is an important process of atherosclerosis. The integrity of the endothelium is maintained by intercellular junctions to prevent vascular leakage (Sluiter et al., 2021). MtROS inhibited by mitochondrion-targeting antioxidant mitoquinone (MitoQ) restore endothelial barrier integrity by preventing VE-cadherin disassembly and actin cytoskeleton remodeling (Chen et al., 2019), which indicate mtROS induce endothelial barrier injury. Increased studies showed that it is through decreasing intercellular junctions to lead to endothelial hyperpermeability (S. Meng S et al., 2022). In H₂O₂-induce endothelial hyperpermeability, cytochrome c release is released from dysfunctional mitochondria, and consequently activation of caspase-3 (Li et al., 2014). Caspase-3 has been shown to cleave β-catenin, thereby disrupting the VE-cadherin-β-catenin complex, which result disruption of cell adherens junctions (Tharakan et al., 2012).

In addition to mentioned above, intracellular mtROS induce endothelial EndMT, senescence to promote atherosclerosis development (Chen C et al., 2021; Jiang et al., 2020; L. Li L et al., 2021; N. Meng N et al., 2022; M. Zhu M et al., 2018).

## Imbalance of endothelial mitochondrial dynamics and atherosclerosis

### Mitochondrial dynamics

**FIGURE 2:**
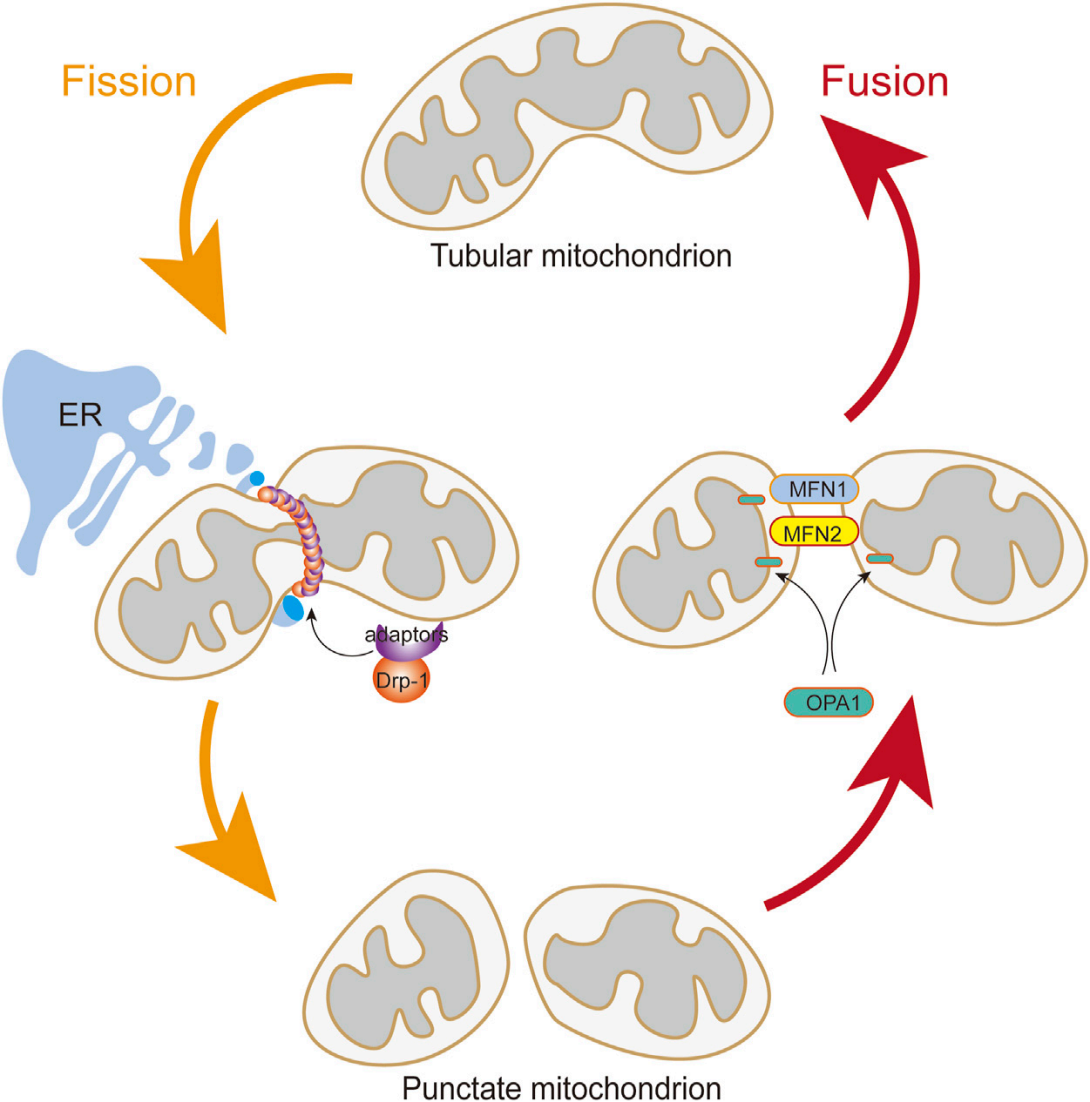
Mitochondrial Fusion and Fission. The translocation of Drp1 to the outer mitochondrial membrane from the cytosol is induced by Drp1 adapters when DRP1 is activated. Assembly of the fission apparatus is in the outer mitochondrial membrane that are aggregated in a microenvironment shaped by contact with the endoplasmic reticulum. Mitochondrial fusion is mediated by the coordinated activities of mitofusins (MFN1 and MFN2) in the outer mitochondrial membrane and OPA1 in the inner mitochondrial membrane.

The mitochondria are extremely dynamic organelles that constantly undergo fusion and fission (Figure 2), and their morphology, number, and size respond rapidly to altered environments through a dynamic network called “mitochondrial dynamics” (Jin et al., 2021; Luan et al., 2021). Mitochondrial dynamics is essential in many cellular processes. However, imbalanced mitochondrial dynamics causes mitochondrial structural alterations and dysfunction. Multiple studies have confirmed the influence of mitochondrial dynamics on vascular diseases (Rao et al., 2020; Wang et al., 2021).

Mitochondrial fusion is controlled by the transmembrane GTPases mitofusin-1 (MFN1) and mitofusin-2 (MFN2) at the outer membrane and by optic atrophy protein 1 (OPA1) at the inner membrane (Li C et al., 2020; Tur et al., 2020). MFN1 and MFN2 are essential for tethering adjacent mitochondria and executing outer membrane fusion by forming antiparallel homodimeric or heterodimeric coiled-coil linkages between adjacent mitochondria (Bockler et al., 2017; Hales and Fuller, 1997). Meanwhile, OPA1 embedded in the inner membrane or intermembrane of the mitochondria is involved in mitochondrial inner membrane fusion and mitochondrial cristae remodeling (Polyakov et al., 2003; Song et al., 2007).

Mitochondrial fission is manipulated by DRP1, fission-1 mitochondrial fission factor (MFF), and mitochondrial dynamic proteins of 49 and 51 kDa (Bockler et al., 2017), among which the DRP1 is the main pro-fission protein with activity, that is, tightly controlled to ensure balanced mitochondrial dynamics (Simula et al., 2019). DRP1 in the cytoplasm executes fission by recruiting to the mitochondrial outer membrane to drive scission (Archer, 2013). DRP1 mediates mitochondrial fission in four distinct steps: transferring from the cytosol to the outer membrane of the mitochondria, incorporating into higher-order complexes with other fission factors, constricting the organelle in a GTP-dependent manner, and ultimately separating the parent organelle into two mitochondria (Ong and Hausenloy, 2017). In addition, DRP1 activity is modulated by two serine phosphorylation sites with opposing functions. DRP1 activity can be reversibly modified by two critical phosphorylation sites. Phosphorylation of DRP1 at serine 616 (p-Drp1S616) promotes DRP1 activity. Conversely, phosphorylation of serine 637 (p-Drp1S637) represses its activity and leads to mitochondrial elongation. Each serine phosphorylation is catalyzed by a different kinase and phosphatase (Cali and Szabadkai, 2018; Ko et al., 2021).

### The vascular risk factors cause imbalanced mitochondrial dynamics in endothelium

The mitochondria undergo a dynamic transition between tubular and fragmented morphologies to respond to cellular energy demands and endogenous and exogenous stressors (Heine and Hood, 2020). An imbalance between fusion and fission can alter mitochondrial morphology. Enhancement of fission or disruption of fusion causes mitochondrial fragmentation. Conversely, enhancement of fusion or disruption of fission results in elongated tubular mitochondria (Chen et al., 2005).

Blocking cytosolic ROS generation or enhancing mitochondrial antioxidant activity prevents mitochondrial fission of the endothelium in hyperglycemia (Bhatt et al., 2013a), which demonstrate that cytosolic and mitochondrial ROS can both enhance mitochondrial fission. ROS or oxidative stress elicit phosphorylation and translocation to the mitochondrial membrane of DRP1 to mediate mitochondrial fission in multiple types of cells (Ni et al., 2020). Increase evidence have explored the regulator for DRP1 phosphorylation such as ERK, PKC and JNK in endothelium response ROS (Chen J et al., 2021; Michalska et al., 2016; Ni et al., 2020). MtDNA damage, including mtDNA mutation and decreased mtDNA copy number, is induced by mitochondrial-derived oxidative stress (Ungvari et al., 2018). MtDNA damages that are not repaired will accumulate and lead to mitochondrial fusion, fission, and mitophagy (Meyer et al., 2017).

Diabetes mellitus is accompanied by high blood sugar levels. Hyperglycemia in patients with diabetes leads to endothelial dysfunction and apoptosis. High-glucose culture conditions significantly alter the mitochondrial morphology in human umbilical vein endothelial cells (HUVECs) (Hao et al., 2019). *In vitro* studies have also implicated that mitochondrial fission is induced by hyperglycemic conditions (Yu et al., 2006). The mechanisms underlying hyperglycemia-induced mitochondrial fission include increased protein expression of DRP1 protein, a key mediator of mitochondrial fission (Hao et al., 2019). However, the more detailed mechanism of DRP1 protein expression increase is still unclear. It has been reported that Rho-associated coiled coil-containing protein kinase 1 (ROCK1) activates DRP1 by phosphorylation at serine 600 residue and promoting DRP1 transfer to mitochondria in hyperglycemia-induced endothelial mitochondrial fission (Wang et al., 2012). However, another study shows ROCK1 as a downstream target of FOXO1 to phosphorylate DRP1 at Ser616 in diabetes-induced endothelial dysfunction (Shi et al., 2018). Furthermore, high-glucose treatment increases cytosolic and mitochondrial ROS in ECs, which exacerbate the progression of hyperglycemia-induced endothelial mitochondrial fission (Bhatt et al., 2013b; Gero et al., 2013; Quagliaro et al., 2003; Querio et al., 2018). Aside from inducing mitochondrial fission, high glucose levels also decrease endothelial mitochondrial fusion by disrupting mitochondrial oxidative phosphorylation (Zeng et al., 2019).

**FIGURE 3:**
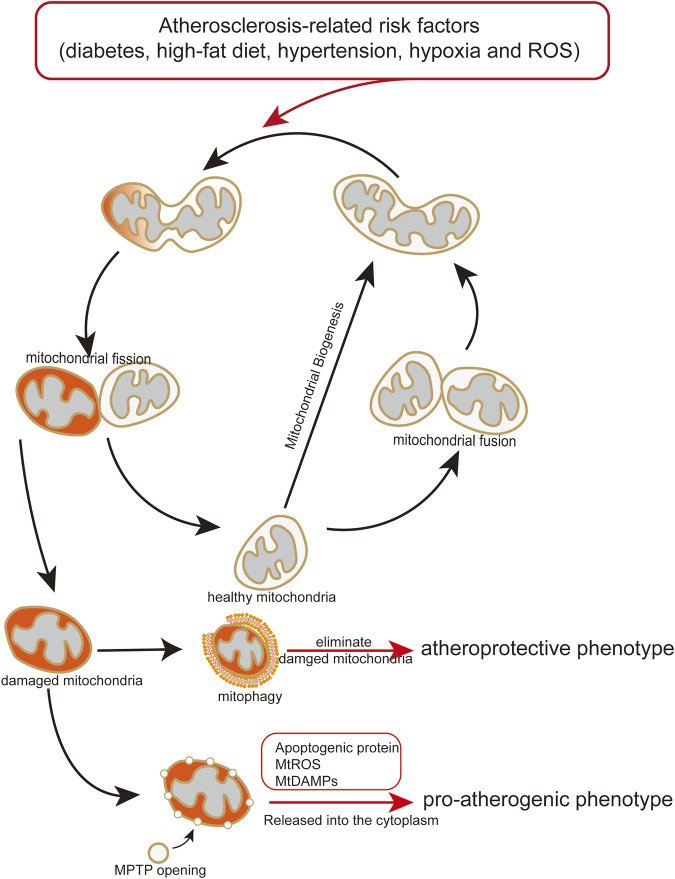
Endothelial mitochondria mediate pro-atherogenic or atheroprotective phenotype. Atherosclerosis-related risk factors induce mitochondrial damage and dysfunction. Damaged or dysfunctional mitochondria release mitochondrial mtDAMPs and ROS to the cytoplasm, causing endothelial transformation to a proatherogenic phenotype. Mitophagy maintains endothelial homeostasis by eliminating damaged or dysfunctional mitochondria, play an atheroprotective role.

Excessive mtROS production is thought to be main reason of ischemia (I)/reperfusion (RP)-induced EC injury. I/RP-induced mtROS production results from increased mitochondrial fission (Giedt et al., 2012b; Scheitlin et al., 2014; Zhou et al., 2017). Disturbed flow, a pro-atherosclerotic blood flow which is implemented through mouse carotid artery ligation and microfluidic experiments, increases mtROS and further induce mitochondrial fission in ECs, whereas unidirectional flow, an atheroprotective blood flow, significantly decreases mitochondrial fragmentation (Hong et al., 2022). Excessive ROS or mtROS can elicit oxidative damage to the mitochondria (Daiber, 2010). In order to control the mitochondria quality, mitochondria isolate the damaged part of mitochondria from the healthy part *via* mitochondrial fission, and the damaged part is eliminated by mitophagy (Figure 3) (Archer, 2013). Besides, ROS production can also promote phosphorylation of DRP1 to mediate mitochondrial fission (Cid-Castro and Moran, 2021).

Ox-LDL is a key risk factor for atherosclerosis. Research have shown that ox-LDL treatment can induce endothelial apoptosis associated with DRP1-related mitochondrial fission (Zheng and Lu, 2020). However, the mechanism underlying ox-LDL-induced mitochondrial fission is still unclear. It is possible that ox-LDL induced mitochondrial fission through excessive cytosolic and mitochondrial ROS. MicroRNA participate in regulation of ox-LDL-induced mitochondrial fission. A-kinase anchoring protein one is a downstream target of microRNA-199b-5p which promoting excessive mitochondrial fission by interaction with DRP1 in ox-LDL-treated HUVECs (Cui et al., 2022). Another microRNA, miR-21-5p, a direct interaction with DRP1, promote ox-LDL-induced endothelial mitochondrial senescence by downregulating the level of DRP1 (Zhang et al., 2017).

LPS induces mitochondrial fission of human lung microvascular ECs by stimulating phosphorylation of DRP1 at S616 and increasing expression of DRP1 (Fu et al., 2021; Lian et al., 2022). Inhibiting activity of DRP-1 or mitochondria fission factor (Mff) attenuated LPS induced mitochondrial fragmentation in primary rat aortic ECs (Forrester et al., 2020). Besides, LPS also induces excessive ROS generation in a time-dependent manner in the mitochondria of human lung microvascular ECs, which can also promote endothelial mitochondrial fission (Fu et al., 2021).

### The imbalance of endothelial mitochondrial dynamics accelerates atherosclerosis

The abnormal dynamic behavior of EC mitochondria may contribute to mitochondrial and EC dysfunction, which is an important hallmark of many vascular diseases, such as atherosclerosis (Giedt et al., 2012a; Hall et al., 2014). Increasing studies have been shown mitochondrial fission mediates endothelial inflammation (Altara et al., 2020; Forrester et al., 2020; Miyao et al., 2020). Nuclear factor (NF)-κB is a master regulator of endothelial inflammation and is strongly y induced by tumor necrosis factor-α (TNF-α) and LPS (M. Li M et al., 2018). A strong causality exists between NF-κB activation and mitochondrial fission in endothelial inflammatory responses (Forrester et al., 2020). TNF-α is a most critical cytokine among several cytokines to induce endothelial inflammation (Karki et al., 2021; Sorokin and Mehta, 2022). It also induces mitochondrial fission by increasing Ser616 phosphorylation of DRP1 in cultured ECs (Forrester et al., 2020; Shen et al., 2018). Genetic inhibition of DRP1 or pharmacological inhibition of mitochondrial fission suppressed TNF-α-induced NF-κB activation, VCAM-1, a downstream target gene of NF-κB, expression and monocyte adhesion (Forrester et al., 2020). In addition, *in vivo*, reduction of DRP1 by genetic manner or a DRP1 inhibitor mdivi-1 also suppress TNF-α–induced leukocyte vascular adhesion. These evidence supports that mitochondrial fission contributes to inflammation by sustained NF-κB activation in ECs (Altara et al., 2020). However, the mechanism underlying mitochondrial fission-mediated NF-κB activation and inflammatory response is still unclear. Mitochondrial fission leads to endothelial oxidative stress due to excessive mtROS production (Yu et al., 2006). NF-κB is a redox-sensitive transcription factor, is upregulated and chronic activated and drives a proinflammatory shift in response oxidative stress (El et al., 2013; Koundouros and Poulogiannis, 2018). This result seems to contradict a previous finding that the SOD2 mimic mitoTempo do not block TNF-α–induced VCAM-1 expression, indicating that ROS is not responsible (Altara et al., 2020), which showed the ROS production accompanied by mitochondrial fission is not the main reason of proinflammatory shift mediated by mitochondrial fission. Thus, other pathways are possibly involved in mitochondrial fission-mediated NF-κB activation and inflammatory response.

Donor microvascular ECs interact with recipient alloreactive memory T cells, which promote responses leading to allograft rejection during cardiac transplantation (Pearl et al., 2005). Research have been shown that inhibiting mitochondrial fission or promoting mitochondrial fusion reduce EC immunogenicity, protect cardiac allografts from injury, and prolong allograft survival (Tran et al., 2022). Increasing endothelial mitochondrial fusion using M1, a pro-mitochondrial fusion molecule, and mdivi1, a DRP-1 inhibitor, reduce the level of TNF-induced ICAM-1 and VCAM-1 expression and the ability to activate co-cultured allogeneic CD8^+^ T cells from a sensitized mouse (Chakraborty et al., 2015). Preventing endothelial mitochondrial fission-induced low ability to activate allogeneic CD8^+^ T cells attribute to diminished expression of cytokine-induced costimulatory molecules and increased EC expression of the T cell inhibitory ligand PD-L1 on ECs (Mullan and Pober, 2022; O'Malley et al., 2018; Tran et al., 2022). Static cold storage and warm reperfusion-induced MPTP opening promotes increased EC immunogenicity (Tran et al., 2018).

Endothelial dysfunction contributes to the development of atherosclerosis in patients with diabetes mellitus (Tabit et al., 2010). Mitochondrial fragmentation and increased expression of fission-1 protein are observed in venous ECs isolated from patients with diabetes mellitus and, human aortic ECs treatment with 30 mmol/L glucose. Mitochondrial fission inhibition by silencing Fis1 or DRP1 expression with siRNA blunted high glucose–induced endothelial dysfunction, which may attribute to decrease of mtROS (Shenouda et al., 2011). Because mitochondrial fragmentation/fission is reqiured for high glucose-induced respiration and excessive ROS production (Yu et al., 2006). ROS scavenger prevents glucose-induced impairment of eNOS activation and cGMP production, representing a restoration of endothelial dysfunction (Shenouda et al., 2011), suggesting that it is through increasing ROS in mitochondrial fission-induced endothelial dysfunction.

High-glucose drive the mitochondrial membrane hyperpolarization of ETC and subsequently increase mtROS production at complexes I and III *via* uncoupled respiration (Brownlee, 2001). It seems that high-glucose-induced mtROS production is independent on mitochondrial fission. However, in hepatocytes, mitochondrial membrane depolarization does not prevent glucose-induced mitochondrial fragmentation but prevents ROS generation (Yu et al., 2006). This suggest that increased mitochondrial ROS is a consequence of mitochondrial fragmentation, not the cause. Scavenging ROS prevent mitochondrial fission in mouse ECs, suggesting that ROS is a trigger for fission under high glucose conditions (Bhatt et al., 2013a). ROS elicit mitochondria oxidative damage and to drive mitochondria isolate the damaged part of mitochondria from the healthy part *via* mitochondrial fission (Archer, 2013; Bhatt et al., 2013b). Thereby, it is necessary to elucidate mitochondrial fission whether mitochondrial fission or ROS is a consequence, cause, or vicious circle in further studies.

Alteration of mitochondrial dynamic by ox-LDL, high glucose or other risk factors of blood vessels result in endothelial dysfunction (Gao et al., 2019; Huang et al., 2018; Li et al., 2015,^2016^), increasing permeability (Fu et al., 2021; She et al., 2021), inflammation (Bhatt et al., 2013a; Hao et al., 2019), apoptosis (Cui et al., 2018) and senescence (Kim et al., 2018; Zhang et al., 2017). In these processes, although the causality between mitochondrial dynamic and ROS production is still unclear, mtROS production that accompany the alteration of mitochondrial dynamic is a key factor for effect of mitochondrial fission on endothelial function and atherosclerotic development.

The vast majority of studies suggest that endothelium is benefit from mitochondrial fusion and inhibition of mitochondrial fission (Duraisamy et al., 2019; Robert et al., 2021; Zeng et al., 2019; Zheng and Lu, 2020). However, another study suggested that mitochondrial fission in heart muscle play a protective role in ischemic-reperfusion induced myocardial infarction (Shimura et al., 2021). Despite the controversy about the effect of mitochondrial fission or fusion, it has been established that an imbalance in mitochondrial dynamics would impair normal endothelial function.

## Endothelial mitophagy and atherosclerosis

### Two group pathways of mitophagy

Autophagy, an evolutionarily conserved mechanism, can arrest superfluous, aging, or damaged cytoplasmic components, including double-membraned organelles, to lysosomes for degradation (Antonioli et al., 2017; Galluzzi et al., 2017). The autophagic system targets impaired mitochondria and delivers them to lysosomes for degradation. This catabolic process, called mitophagy, is a fundamental mechanism that regulates mitochondrial quality and quantity control (Onishi et al., 2021; Pickles et al., 2018). Mitophagy includes six steps: 1) isolation of impaired mitochondria *via* fragmentation, 2) activation of mitophagy receptors on the mitochondrial surface or recruitment of ubiquitin–autophagy adaptors to the surface of mitochondria, 3) autophagy proteins target the mitochondria and form an isolation membrane around the organelle (phagophore). 4) formation of autophagosomes, 5) fusion of autophagosomes with lysosomes, and 6) mitochondria are degraded into lysosomal acidic hydrolases, and products of dissociation are recycled (Onishi et al., 2021).

In mammals, mitophagy occurs in two pathways: ubiquitin-mediated mitophagy and receptor-mediated mitophagy. Mitophagy is regulated by the outer mitochondrial membrane kinase PINK1 and the cytosolic E3 ubiquitin ligase Parkin in ubiquitin-mediated mitophagy (Matsuda et al., 2010; Narendra et al., 2010,^2008^). In mitochondrial damage, Parkin translocation from the cytosol to damaged mitochondria promotes the ubiquitination of several mitochondrial outer membrane proteins. The participation of PINK1 is required for Parkin activation and recruitment to damaged mitochondria. PINK1-mediated phosphorylation plays an important role in Parkin activation (Shiba-Fukushima et al., 2012). PINK1 directly phosphorylates Parkin on Ser65, and the site phosphorylation is required for the ubiquitin activity of Parkin (Kondapalli et al., 2012). In healthy mitochondria, PINK1 is maintained at low levels due to post-transcriptional regulation and rapid degradation by proteolysis. When the mitochondria are damaged, PINK1 degradation is inhibited, leading to PINK1 accumulation within the damaged mitochondria (Vasquez-Trincado et al., 2016). Thus, Parkin is recruited to the mitochondria and ubiquitylates the proteins, such as mitofusin, Miro, and VDAC, on the mitochondrial surface (Gegg et al., 2010; Tanaka et al., 2010; Wang et al., 2011). PINK1- and Parkin-catalyzed high-level ubiquitylation facilitates mitophagy through phagophore formation (Okatsu et al., 2015). Then, whole autophagosomes are formed and fuse with lysosomes (Antonioli et al., 2017). PINK1-Parkin pathway-mediated mitophagy is first observed in Parkinson’s disease (Lotharius and Brundin, 2002).

In receptor-mediated mitophagy, two major types of receptors have been suggested to mediate the elimination of damaged mitochondria. One group is BNIP3 and BNIP3L, and another group is FUNDC1 (Chen et al., 1999; Hanna et al., 2012). BNIP3 is required for the mitochondrial turnover under hypoxic conditions (Zhang et al., 2008). In healthy conditions, BNIP3 is usually expressed in the cytosol and is an inactive monomeric formation (Fu et al., 2020). It forms a stable homodimer following stress signals and translocates to the outer mitochondrial membrane (Fu et al., 2020; Kubli et al., 2008). BNIP3 has a LIR motif at its N-terminal region interaction with LC3, leading to mitophagy (Zeng et al., 2021). NIX is a homology of BNIP3, promoting the selective degradation of mitochondria (Da et al., 2021). Similar to BNIP3, NIX also has a LIR motif at its N-terminal region interaction with LC3, and functional dimerization is regulated by phosphorylation (Lampert et al., 2019; Rogov et al., 2017). Another receptor is FUNDC1, an outer mitochondrial membrane protein. FUNDC1 expression is decreased in a ubiquitin–proteasome-dependent manner because of the March5-mediated ubiquitylation of FUNDC1 at Lys119 during hypoxia (Chen et al., 2017). Thus, FUNDC1 degradation by endogenous March5 desensitizes mitochondria to hypoxia-induced mitophagy (Chen et al., 2017). FUNDC1 also contains a typical LIR motif at the N-terminal region (Liu et al., 2012). FUNDC1 interact with LC3 on the LIR motif, which is regulated *via* phosphorylation and dephosphorylation on residues Ser13 and Tyr18 of the LIR motif (Liu et al., 2012,^2021^).

OPA1, which is coded by a nuclear gene and located on the inner mitochondrial membrane, is a key regulator of the balance between mitochondrial fusion and fission (Li M et al., 2020; Vasquez-Trincado et al., 2016). Reduction in mitochondrial fusion in mouse ECs by EC-specific Opa1 knockout promotes atherosclerotic development in Ldlr^−/−^mice, providing direct evidence for mitochondrial dynamics mediating atherosclerotic development (Chehaitly et al., 2022). The endothelial OPA1 expression level is reduced by HFD *in vivo* and ox-LDL *in vitro*. Coenzyme Q10 promote endothelial OPA1 expression by AMPK-YAP axis, and alleviating atherosclerosis in HFD fed ApoE^−/−^ mice (Xie et al., 2020). Anti-diabetic drugs play an anti-atherosclerotic role *via* the AMPK-mediated blockage of DRP1-mediated mitochondrial fission in ECs of diabetic ApoE^−/−^/AMPKα2^−/−^ mice (Wang et al., 2017). Gypenoside, an extraction product of Gynostemma pentaphyllum, inhibits atherosclerotic plaques and thickening of the aortic intima in ApoE^−/−^ mice. The anti-atherosclerotic effect of gypenoside is mediated by mitochondrial fission and fusion proteins inhibiting endothelial apoptosis (Song et al., 2020). These studies suggest that atherosclerosis development can be mediated by endothelial mitochondrial dynamics.

### The atheroprotective role of mitophagy in endothelial cells

In ECs, mitophagy plays an atheroprotective role. Vascular risk factor-induced EC injury, dysfunction, or death can be alleviated by mitophagy (C. Chen C et al., 2022; Li C et al., 2020; Xi et al., 2021; Xiang et al., 2022). Under risk factors-induced stress, the mitochondria in ECs show dysfunction, including increased ROS production, opening of the mitochondrial permeability transition pore, release of mitochondrial mtDAMPs and other mitochondria-derived peptides, decreased mitochondrial membrane potential and elevated caspase-3/9 activity (C. Chen C et al., 2022; Huang et al., 2020; Shimada et al., 2012; Zheng and Lu, 2020). This event ultimately leads to inflammation and damage of the intima, the main cause of atherosclerotic initiation (Qian et al., 2021). Mitophagy specifically eliminates damaged or dysfunctional mitochondria, which partly prevents endothelial disorder, suggesting the potential atheroprotective role of this process (Fan et al., 2022; Li et al., 2022; Zhang et al., 2022).

Enhancing mitophagy by resveratrol (Li C et al., 2020), brain-derived neurotrophic factor (Jin et al., 2019), and scutellarin (Xi et al., 2021) alleviate risk factor-induced negative effects in ECs *via* the upregulation of proteins associated with mitophagy. Resveratrol, a stilbenoid, can enhance BNIP3-mediated mitophagy which prevent ox-LDL-mediated mitochondrial dysfunction, including mitochondrial respiration complexes inactivation, sustaining mitochondrial membrane potential, and consequently favoring EC survival. At the molecular level, resveratrol treatment accompanies with an increase of HIF1 and AMPK levels and promoting BNIP3 transcription and expression, consequently enhancing BNIP3-mediated mitophagy (Li C et al., 2020). Mature brain-derived neurotrophic factor (BDNF) plays a protective role against high-glucose treatment-caused endothelial dysfunction *via* inducing BNIP3-mediated mitophagy. BDNF treatment exhibit increased LC3-II protein levels and decreased p62 levels, indicating that BDNF enhance autophagy flux. BDNF binding to TrkB, a high affinity receptor of BDNF, trigger mitophagy through the HIF-1α/BNIP3 signaling pathway (Jin et al., 2019). Scutellarin, a plant extract, upregulate mitophagy *via* PINK1/Parkin signal pathway against hyperglycemia-induced endothelial injury (Xi et al., 2021).

### Mitophagy activation by atherosclerotic risk factors in endothelial cells

Exposure of ECs to vascular risk factors leads to mitochondrial damage, such as endothelial mtROS overproduction, mitochondrial membrane potential reduction, mitochondrial fission, and mitochondrial dysfunction (Li C et al., 2020; Xie et al., 2021). These then trigger intracellular stress response, resulting in EC injury or dysfunction, consequently promoting the development of atherosclerosis (P. Li P et al., 2018; Wang et al., 2020). Following mitochondria damage, mitophagy removes damaged and dysfunctional mitochondria to maintain intracellular homeostasis in the cardiovascular system (Y. Chen Y et al., 2022; He et al., 2019; Huynh and Heo, 2021; Tu et al., 2022; Yang et al., 2022).Mitophagy-mediated elimination of damaged mitochondria alleviates mitochondrial damaged-induced EC injury or dysfunction (Bhogal et al., 2018; Zekri-Nechar et al., 2022).

Exposure of ECs to high glucose levels not only induces excessive mitochondrial fragmentation and ROS generation but also decreases mitophagy, which accelerates dysfunctional mitochondrial accumulation (W. Zhu W et al., 2018). Defective mitophagy is observed in ECs from diabetic rats. High glucose condition reduces mRNA and protein levels of Pink1, Parkin, and LC3B in ECs, suggesting an inhibition of mitophagy (C. Chen C et al., 2022; W. Zhu W et al., 2018). These studies show that high glucose levels inhibit mitophagy in ECs by reducing the expression levels of ubiquitin-mediated mitophagy proteins.

Aortic ECs isolated from mice fed a high-fat diet or treated with oxidized low-density lipoprotein (100 μM) evoke excessive mitophagy mediated by Parkin. At the molecular level, ox-LDL stimulation increases NR4A1 expression, which induces the phosphorylated activation of Parkin by activated CaMKII (P. Li P et al., 2018). Contrary to the effect of high glucose levels on mitophagy, high fat induces excessive mitophagy and leads to endothelial apoptosis (P. Li P et al., 2018). However, cardiac mitophagy has been observed at 3 weeks and lasts after 2 months in mice fed a high-fat diet, suggesting that high fat-induced mitophagy plays a protective role against obesity cardiomyopathy (Tong et al., 2019). However, some studies reported that ox-LDL impairs mitophagy by regulating the expression of mitophagy markers *via* the PTEN-Mfn2 axis (Li P et al., 2020), and resveratrol reduces hyperlipemia-induced endothelial damage by enhancing BNIP3-related mitophagy (Li C et al., 2020). These contradictions may be ascribed to spatial and temporal differences.

ROS-triggered oxidative damage increases mitochondrial ROS production and the disturbance of mitochondrial function, leading to parkin-1-mediated mitophagy in brain ECs (D. Kim D et al., 2020). Intracellular ROS promotes LC3B to co-localize with the mitochondria in liver ECs during I/RP injury (Bhogal et al., 2018), suggesting an increase of mitophagy. ATG7 activation is upstream LC3B lipidation and autophagosome formation (Noda and Inagaki, 2015; Galluzzi and Green, 2019). ROS inhibition by N-acetylcysteine reduces the activity of ATG7 but does not affect ATG5, Beclin-1, or ATG12, suggesting that ROS-ATG7 axis y is an important mechanism for I/RP injury-induced liver endothelial mitophagy (Bhogal et al., 2018). ROS upregulates the expression of p66Shc, an oxidoreductase that produces ROS in a mitochondria-dependent manner (Boengler et al., 2019; Piao et al., 2020; Y. Zhao Y et al., 2019). p66shc knockdown by siRNA transfection suppresses the mRNA expression of mitophagy markers (LC3, PINK1, and parkin) in HUVECs, suggesting that p66shc is required for ROS-induced mitophagy (Piao et al., 2020).

Mitochondrial autophagy is induced by hypoxia possibly through the hypoxia-dependent factor-1-dependent expression of BNIP3 (Zhang et al., 2008). Hypoxia strongly induces the expression of BNIP3, which is a target gene of HIF-1 (Kubasiak et al., 2002; Mazure and Pouyssegur, 2009). Hypoxia anti-VEGF agent bevacizumab in HUVECs induces mitophagy activating the HIF-1α-BNIP3/FUNDC1 signaling pathway. These results suggest that hypoxia-induced mitophagy plays a protective role against hypoxia by maintaining mitochondrial quality, sustaining metabolic homeostasis, and reducing ROS generation (Sun et al., 2021).

As discussed above, mitochondrial autophagy is induced by high glucose levels, high-fat diet, ROS, hypoxia, and other risk factors of blood vessels. In theory, the factors causing mitochondrial damage can also trigger mitophagy to maintaining mitochondrial quality and metabolic homeostasis. However, if activated mitophagy is not enough to elimination of damaged mitochondria, which would lead to endothelial disorder and consequently promoting development of atherosclerosis (Figure 3).

## Other factors from endothelial mitochondria and atherosclerosis

DAMPs are traditionally thought to trigger inflammation through release into the extracellular environment. Recent finding shave shown mitochondria-derived damage-associated molecular patterns (mtDAMPs) in triggering sterile inflammation (Nakahira et al., 2015). MtDAMPs include mtDNA, cytochrome C, cardiolipin, heat shock protein 60 (HSP60), mitochondrial transcription factor A, and N-formyl peptides (Khwaja et al., 2021). The role of mtDAMPs in ECs has been investigated. MtDNA can been released into the cytoplasm from damaged mitochondria by MPTP (Nakahira et al., 2015). Then mtDNA is recognized by TLR9, and triggering endothelial inflammatory response (Yu and Bennett, 2016). In addition, mtDNA and peptides lead to pathologic endothelial permeability through neutrophil-dependent and -independent pathways (Sun et al., 2013). Another mtDAMP, oxidized cardiolipin (oxCL), recruits additional monocytes to the intimal layer by increasing the expression of ICAM-1 and VCAM-1 on the EC membrane, suggesting that it plays a pro-inflammatory role (Khwaja et al., 2021). Hydrolysis of oxCL-produced lysoCL and oxidized octadecadienoic acid metabolites impair pulmonary endothelial barrier function (Buland et al., 2016), whereas inhibition of the oxidation of endothelial CL reduces these effects (Liu et al., 2014; Wan et al., 2014). HSP60 is upregulated in the arterial ECs by risk factors associated with atherosclerosis, resulting in cell death through binding to TLR4/CD14, thereby promoting the NF-κβ pathway (Xu et al., 2000).

Given a bad reputation and mitochondrial evolutive homology with bacteria, mtDAMPs are expected to play proatherogenic roles (Khwaja et al., 2021). The relationship of mtDAMPs with atherosclerosis has been extensively investigated in several types of cells associated with atherosclerosis, including monocytes (Dela and Kang, 2018), vascular SMCs (Yu et al., 2017) and ECs (Huang et al., 2020; Zhang et al., 2010). Most studies focused on atherosclerotic intraplaque cells, which mainly consist of macrophages and SMCs, because of the huge inventory of mtDAMPs in plaque (Yu et al., 2013). However, ECs are more vulnerable than other cells because they are stimulated by mtDAMPs from themselves and circulation in the initiation step of atherosclerosis (Alvarado-Vasquez, 2015; Faust et al., 2020; Qian et al., 2021). Increasing evidence show that mtDAMPs promote endothelial transformation to a proatherogenic phenotype by triggering inflammatory response, which is a key step for atherosclerotic initiation (Khwaja et al., 2021; Qian et al., 2021; Salnikova et al., 2021).

Coupling factor 6 (CF6), a mitochondria-derived peptide located on the EC surface and mitochondria, can be released from the extracellular space of ECs (Osanai et al., 2001). CF6 in ECs has been associated with inflammation. TNF-a can stimulate the movement of CF6 from the mitochondria to the extracellular space in ECs *via* the NF- κB pathway (Sasaki et al., 2004). Application of CF6 reduces shear stress-induced NO release by downregulating PECAM-1, implying a pro-atherogenic a role (Kumagai et al., 2008).

Other mitochondria-derived peptides, humanin and prohibitin-1, are suggested to promote EC survival by reducing the oxidized low-density lipoprotein-induced formation of ROS and apoptosis (Oh et al., 2011; Schleicher et al., 2008).

## Conclusion and perspectives

This review suggests that mitochondrial damage and dysfunction affect endothelial function and trigger the initiation and progression of atherosclerosis (Table 2) (Hall et al., 2014). Following mitochondrial damage and dysfunction, mitophagy plays a pivotal role in maintaining endothelial homeostasis by eliminating damaged or dysfunctional mitochondria, which can decrease the release of mitochondrial mtDAMPs and ROS to the cytoplasm from damaged or dysfunctional mitochondria, and attenuating endothelial disorder (Figure 3) (Y. Chen Y et al., 2022; Zekri-Nechar et al., 2022; Zhang et al., 2008). Mitochondrial dysfunction induced by atherosclerosis-related risk factors in ECs is an important mechanism of atherosclerotic initiation and progression. Considering the crucial role of the mitochondria in endothelial and vascular homeostasis, we recommend promoting mitophagy and maintaining mitochondrial dynamics as potential therapeutic strategies for atherosclerosis.

**TABLE 2:** Mitochondria-mediated endothelium disorder.

Types	Mitochondria-derived inducers or mitochondrial action	Pathways/References	
Endothelial dysfunction	excessive mtROS production	Uncoupling of endothelial nitric oxide synthase (eNOS)	Cassuto et al. (2014); Diers et al. (2013); Radi (2018)
	mtDAMPs	Inflammation	Nakahira et al. (2015); Yu and Bennett (2016)
	CF6	Reducing NO release	
Endothelial cell apoptosis	excessive mtROS production	Apoptogenic protein release	Papu et al. (2019); Salnikova et al. (2021); Wei and Lee (2002)
	Inhibiting mitochondrial fusion	Unknown	Zheng and Lu, (2020)
	excessive mitochondrial fission	Excessive mtROS production	Altara et al. (2020); Forrester et al. (2020); Miyao et al. (2020)
	MPTP openings	Apoptogenic protein and mtDAMPs release	Papu et al. (2019); Wei and Lee (2002)
Endothelial inflammatory responses	mtDNA	TLR9-mediated inflammatory responses	Nakahira et al. (2015); Yu and Bennett (2016)
	oxCL	Expression of ICAM-1 and VCAM-1	Khwaja et al. (2021)
		Recruitment of monocytes	Khwaja et al. (2021)
	CF6	Activating NF- κB pathway	Xu et al. (2000)
EC immunogenicity	Inhibiting mitochondrial fusion	Diminishing costimulatory molecules expression and increasing T cell inhibitory ligand PD-L expression	Mullan and Pober (2022); Tran et al. (2022)
	MPTP opening	MHC-I Antigen-Presenting Machinery Expression	Tran et al. (2018)
		Adhesion and MHC-I Surface Expression	
Endothelial permeability	mtDAMPs	Neutrophil -EC interactions	Buland et al. (2016)
	Complex I blockade	Decreasing ATP generation	Bongard et al. (2013)
Endothelial senescence	excessive mtROS production	Oxidative damage	Chang et al. (2020); S. Kim S et al. (2020)
		Activating NF- κB inflammasome	
	Mitochondrial Fission	Inducing mtROS production	Kim et al. (2018); Zhang et al. (2017)
